# A comparison of dose distributions in gross tumor volume between boron neutron capture therapy alone and combined boron neutron capture therapy plus intensity modulation radiation therapy for head and neck cancer

**DOI:** 10.1371/journal.pone.0210626

**Published:** 2019-04-04

**Authors:** Jia-Cheng Lee, Keh-Shih Chuang, Yen-Wan Hsueh Liu, Tzung-Yi Lin, Yi-Chiao Teng, Ling-Wei Wang

**Affiliations:** 1 Department Oncology, Taipei Veterans General Hospital, Taipei, Taiwan; 2 Department of Biomedical Engineering and Environmental Sciences, National Tsing Hua University, Hsinchu, Taiwan; 3 Institute of Nuclear Engineering and Science, National Tsing Hua University, Hsinchu, Taiwan; 4 National Yang-Ming University, Taipei, Taiwan; Northwestern University Feinberg School of Medicine, UNITED STATES

## Abstract

Nine patients with recurrent head and neck (H&N) cancer received boron neutron capture therapy (BNCT) in one fraction at the Tsing-Hua Open pool reactor (THOR) utilizing the THORplan treatment planning system (TPS). The aims of the present study were to evaluate the use of intensity modulated radiation therapy (IMRT) of 45 Gy in 20 fractions to compensate for the dose heterogeneity in gross tumor volume observed with single-fraction BNCT with mean prescription dose 19 Gy (w), and to evaluate planning quality indices of simulated BNCT+IMRT versus single-fraction BNCT alone. All IMRT plans were generated using the Eclipse TPS which employs the analytical anisotropic algorithm. The conformity index for the gross tumor volume (GTV) was better for the BNCT+IMRT plan than for the BNCT-alone plan (*p* = 0.003). In addition, the BNCT+IMRT plan provided significantly better homogeneity in the GTV (*p* = 0.03). The cold spots in inhomogeneous dose distribution in the BNCT plan may be a key factor for H&N cancer recurrence. Our results suggest that single-fraction BNCT combined with compensated multi-fraction IMRT improves treatment homogeneity and conformity than single-fraction BNCT alone, especially for tumor volumes >100 cm^3^, and possibly increases local tumor control.

## Introduction

In 2014, more than 7000 patients in Taiwan were diagnosed with head and neck (H&N) cancer [1]. Local recurrence of H&N cancer after multidisciplinary treatment, such as surgery combined with radiotherapy and chemotherapy, is not uncommon. The treatment of recurrent H&N cancer is challenging, as the normal tissue has received photon radiation approaching the dose limit and re-irradiation may be associated with a high complication rate. From literature review, intensity modulated radiation therapy (IMRT) was used for salvage therapy of recurrent H&N cancer with diverse toxicity rate (including grade 5 toxicity); and 2-year local control rate ranged from 41 to 58% according to different studies [2–4]. Their results were usually unsatisfactory and this group of patients needs more treatment options.

**Fig 1:**
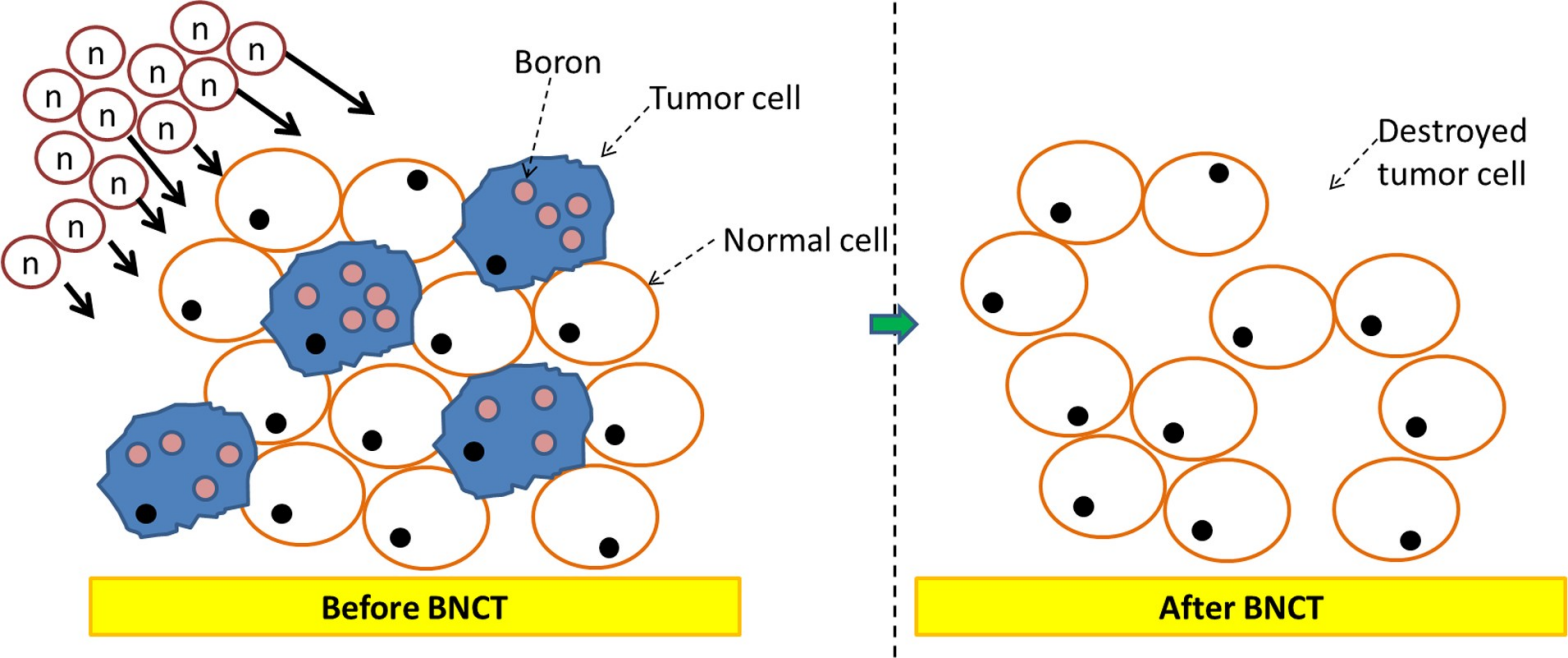
BNCT could selectively damage tumor cells while sparing normal ones as a targeted radiotherapy.

Boron Neutron Capture Therapy (BNCT), which enables selective irradiation at the cellular level, has been used to treat locally recurrent H&N cancer, with high reported response rates (60–83%) [5, 6]. BNCT is a unique type of radiation therapy that enables targeting of cancer at the cellular level (Fig 1) and the neutron-capture reaction (^10^B(n,α)^7^Li) produces alpha particles and Lithium nuclei with high linear energy transfer (LET).

$$\begin{array}{l} {}^{10}B+n\to {}^{11}B\to \{\begin{array}{c} {}^{4}H+{}^{7}Li+2.79\;MeV\;(6\%)\\ {}^{4}H+{}^{7}Li+2.31\;MeV\;(94\%) \end{array}\\ \;↓\\ \;{}^{7}Li+\gamma +0.48\;MeV \end{array}$$

Theoretically, BNCT might procure less treatment toxicity than IMRT because the former is an intra-cellular target radiotherapy and normal structures are better spared by BNCT. However, no randomized clinical trial was performed to fairly compare these two modalities up to now.

In 2008, the National Tsing-Hua University and Taipei Veterans General Hospital initiated a cooperative effort to develop a clinical trial protocol for recurrent H&N cancer involving BNCT. A protocol for fractionated BNCT performed at the Tsing-Hua Open pool reactor (THOR) had been developed by 2010 [7], and we have published several reports describing the treatment results [7–9]. An in-house designed treatment plan (THORplan) was designed for BNCT treatment planning [10–14].

Despite the initially high response rate (70.6%), local recurrence of H&N cancer after BNCT is commonly observed [6, 7, 9]. Our 2-year local recurrent rate was 72%. This might be related cold spots of dose distribution, which may result from insufficient penetration of epithermal neutrons at greater tumor depths and imhomogeneous boron drug absorption. Kawabata et al. [15] reported that the application of an additional three dose gradient (8, 16, and 24 Gy) of X-ray radiation therapy (XRT) to BNCT prolonged the median survival time of 9.4 months in patients with newly diagnosed glioblastoma who were treated with temozolomide. Image-guided IMRT (IG-IMRT) has been used in our hospital for some selected recurrent H&N cancer since 2012. We thought combination of IG-IMRT with BNCT might treat these patients better than Kawabata’s method.

In 2014, a second clinical trial protocol for combined single-fraction BNCT and IG-IMRT was established for recurrent H&N cancer. We hypothesized that the reduced dose heterogeneity of BNCT and higher tumor dose would improve local tumor control relative to pure BNCT. Our clinical trial was approved by the IRB of our hospital with approval No. 2012-06-016A#7.

The purposes of the present study were to explore the feasibility of generating ideal plans combining multi-fraction IMRT and single-fraction BNCT, and to evaluate planning quality indices for simulated BNCT+IMRT versus single-fraction BNCT alone.

## Materials and methods

### Patient selection

For this study, nine patients with recurrent H&N cancer underwent simulated computed tomography (CT) simulation according to the standard protocol of Taipei Veterans General Hospital for BNCT. Four patients had oral cavity cancer, two had oropharyngeal cancer, and one patient each had mandibular sarcoma, a parotid gland tumor and hypopharyngeal cancer. The median accumulated radiotherapy photon dose before BNCT was 66 (range, 60–102) Gy. CT data were acquired with a simulator (HiSpeed; GE Healthcare, Waukesha, WI, USA) according to our institution’s standard protocol, which included the collection of 5-mm slice thicknesses with a 38.4-cm field of view while patients were in the supine position. All patients provided written consent for storage of their medical information in the hospital database and for research use. The study has been approved by Institutional Review Board, Taipei Veterans General Hospital No.2012-06-016A#7 and conducted according to the principles expressed in the Declaration of Helsinki. The individuals in this manuscript (or their relative) have given written informed consent to publish the case details and images.

### Treatment planning process

The patients also received BNCT at the THOR according to the THORplan treatment planning system (TPS) with a Monte Carlo N-Particle Transport Code calculation core. Based on the results of the Monte Carlo calculations, we used compound biological effectiveness (CBE) factors of 3.8, 4.9, 2.5, and 1.3 for p-boronophenylalanine (BPA) in tumor cells, mucosa, skin, and other normal tissue, respectively, to compute total biologically weighted doses [16]. A relative biological effectiveness (RBE) factor of 3.2 was used for THOR epithermal neutron beam high linear energy transfer components, such as the products of thermal neutron capture in nitrogen and fast neutrons; and RBE of 1.0 was used for photons. In BNCT, the total weighted dose (*Gy*(*w*)) was derived from equation:

$$D_{w}=w_{B}\times D_{B}+w_{n}\times D_{n}+w_{\gamma }\times D_{\gamma }$$

where *D*_*B*_, *D*_*n*_, and *D*_*γ*_ are boron dose, neutron dose, and gamma-ray dose, respectively. The *w*_*B*_ is CBE value of boron dose, and *w*_*n*_, and *w*_*γ*_ are RBE values of neutron dose, and gamma-ray dose, respectively.

After BNCT, the same CT images were used to develop photon IMRT plans with Eclipse software (ver.13.0; Varian Medical Systems, Palo Alto, CA, USA) using the analytical anisotropic algorithm (AAA).

An experienced oncologist determined the gross tumor volume (GTV) and organs at risk (OAR) such as the spinal cord, parotid gland, eyes, and mandible, using fluorine-18-labeled BPA positron emission tomography, magnetic resonance, and CT images. In this simulation planning, we assumed that the clinical target volume was equal to the GTV. A safety margin of 3 mm, constrained to 2 mm behind the skin contour, was added automatically to each GTV to create a planning target volume (PTV). In BNCT planning, a single field was set to cover the GTV with the shortest depth, based on the CT images. The principle of prescription dose was 18 Gy (w) to 80% of the GTV in a single fraction. The prescription dose could be adjusted according to the dose limit for normal tissue. All IMRT plans were generated using the Eclipse TPS with the AAA, and the IMRT optimization procedure was based on BNCT-alone plan. Simulated total doses of 65 Gy to the GTV and 45 Gy to the PTV, applied in 20 fractions with six step-and-shoot IMRT fields, were planned. Because the RBE value for photons is 1, we converted the physical dose (Gy) from the IMRT plan to the biological dose (Gy (w)) by multiplying by this value. BNCT and IMRT details for all patients, including prescribed doses (PDs), number of fractions, dose per fraction, GTV, and number of fields, are presented in Table 1. The BNCT plans were used in our clinical trial.

**Table 1:** BNCT and IMRT plan details for the study cohort.

Patient no.	V_GTV_ (cm^3^)	BNCT plan			IMRT plan		
		PD for GTV (Gy (w))	Mean GTV dose (Gy (w))	Beam arrangement (°)	PD for PTV (Gy (w)) in 20 fractions	Mean GTV dose per fraction (Gy (w))	Beam arrangement (°)
1	8.4	18.0	17.7	45	45	2.5	110, 60, 20, 340, 300, 250
2	273.7	18.0	31.3	90	45	2.1	340, 20, 60, 100, 140, 179
3	37.8	20.0	22.5	270	45	2.3	30, 350, 310, 270, 230, 200
4	6.1	20.0	21.2	90	45	2.4	350, 25, 60, 95, 130, 165
5	276.5	17.0	25.3	270	45	2.3	350, 315, 280, 245, 210, 181
6	4.5	18.7	21.6	270	45	2.3	340, 310, 280, 250, 220, 190
7	13.5	14.9	17.9	270	45	2.5	340, 310, 280, 250, 220, 190
8	51.2	21.8	29.2	90	45	2.0	350, 20, 50, 80, 110, 140
9	54.5	23.3	25.0	270	45	2.2	350, 320, 290, 250, 220, 190

BNCT, boron neutron capture therapy; IMRT, intensity-modulated radiation therapy; V_GTV_, volume of gross tumor volume; PD, prescribed dose; GTV, gross tumor volume; PTV, planning target volume.

A linear accelerator with a 6-MV photon and 120 leaves (Clinac iX Millennium 120; Varian Associates, Palo Alto, CA, USA) was used. Optimization was performed to obtain the best plan for each technique and individual patient, and the best BNCT plan was determined prior to IMRT optimization. The interval between BNCT and IMRT was chosen to allow for normal tissue repair. In practice, IMRT began approximately 1 month after the completion of BNCT. Ideally, at least 100% of the IMRT PD was applied to 97% of the GTV, and at least 95% of the PD was applied to 95% of the PTV, while minimizing the volume that received ≥ 110% of the dose. Initially, the planning objectives for the OAR included the application of mean doses < 26 Gy (w) to the parotid gland, and < 42 Gy (w) to the submandibular gland, and maximal doses < 20 Gy (w) to the spinal cord, < 30 Gy (w) to the eyes, < 5 Gy (w) to the eye lens, and < 65 Gy (w) to the mandible.

### Dosimetric evaluation

Planning quality indices based on cumulative dose volume histograms, including conformity index (CI) and homogeneity index (HI) values for the GTV in the BNCT and BNCT+IMRT plans, were evaluated. The CI and HI for the GTV were calculated for each plan according to radiation therapy oncology group definitions [17]:

$$CI=\frac{V_{PD}}{V_{GTV}}\;\mathrm{and}$$

$$HI=\frac{D_{2\%}}{PD\;of\;GTV}$$

where V_PD_ is the volume of the prescription isodose surface, V_GTV_ is the volume of the GTV, and D_2%_ is the maximal dose (Gy (w)) received by 2% of the GTV. The mean dose, minimum dose, and relative volumes greater than V_110_, V_95_, and V_90_ of the PDs for both target volumes were also compared [18, 19]. To account for treatment efficiency, the estimated BNCT and IMRT beam-on times (calculated from the planning system for a maximal dose rate of 600 MU/min for IMRT and 1.2 MW power for BNCT) were recorded and analyzed for the BNCT and BNCT+IMRT plan.

The mean dose, D_2/3V_ (Gy (w)), and D_1/2V_ (Gy (w)) for the parotid gland, maximum significant dose and physical maximum point dose for the spinal cord [20], mean dose and physical maximum point dose for the eyes, and physical maximum point dose for the mandible were determined. The maximum significant doses was defined by International Commission of Radiation Units and Measurements (ICRU) 50 report [21], and the volume defining a significant region was equivalent to a sphere with a radius of 0.75 cm [22].

To score for the PD of the PTV outside the PTV in the BNCT+IMRT plan, and low dose spillage outside the GTV in the BNCT and BNCT+IMRT plans, two dose spillage indices (DSIs) were used [23]:

$${DSI}_{45}=\frac{V_{45}}{V_{PTV}}\;\mathrm{and}$$

$${DSI}_{5}=\frac{V_{5}}{V_{GTV}}$$

where V_45_ and V_5_ are the volumes outside the PTV and GTV receiving ≥ 45 Gy (w) and ≥ 5 Gy (w), and V_PTV_ and V_GTV_ are the volumes of the PTV and GTV, respectively.

### Statistical analysis

All statistical tests were performed using SPSS software (version 20.0; SPSS Inc., Chicago, IL, USA). The paired *t*-test was used to compare dosimetric differences between the BNCT and BNCT+IMRT plans. All tests were two-sided and differences were considered to be significant when *p* ≤ 0.05.

## Results

**Fig 2:**
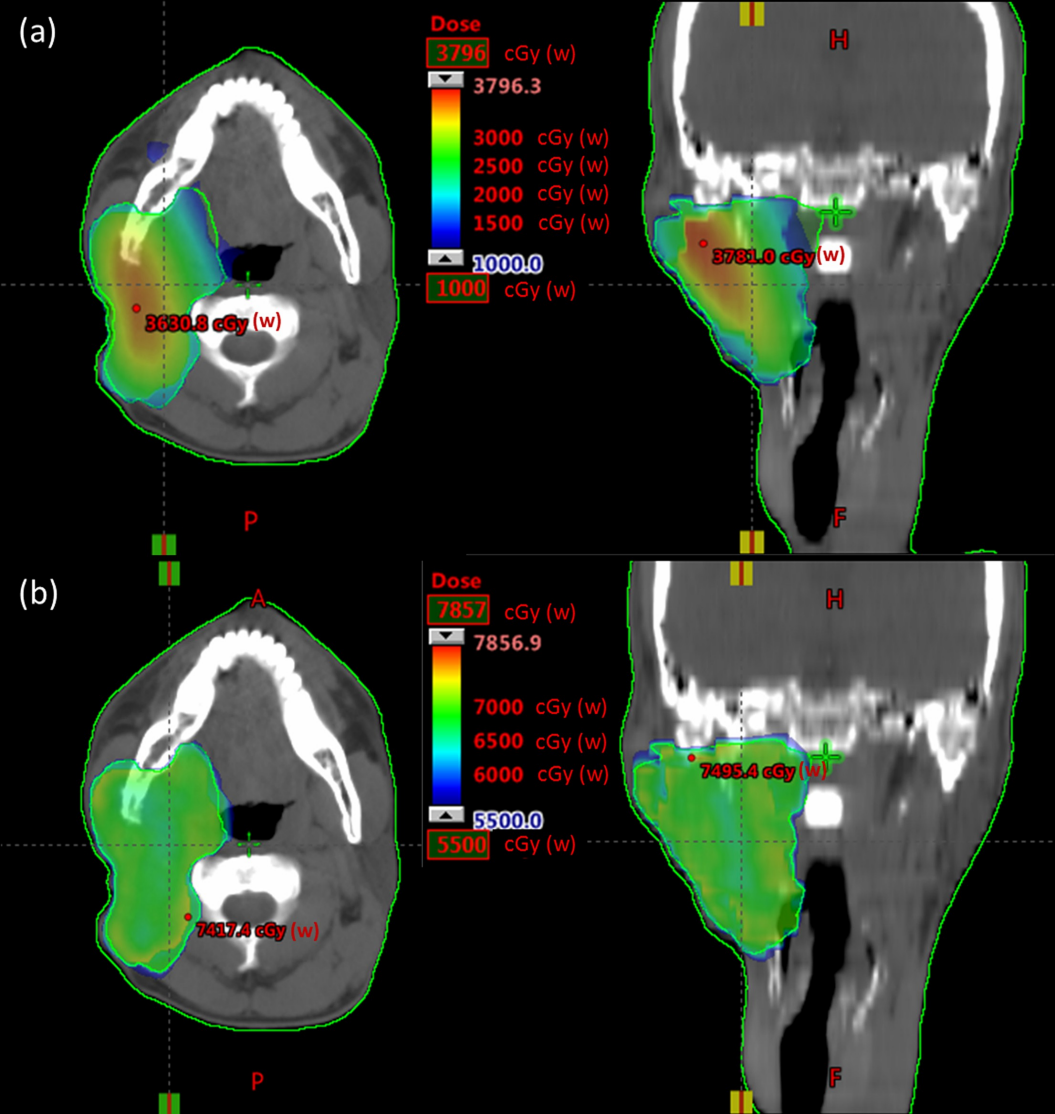
Representative isodose curves of GTV for the BNCT alone (a) in axial and coronal views and BNCT+IMRT (b) plans in axial and coronal views for a recurrent parotid gland tumor.

**Fig 3:**
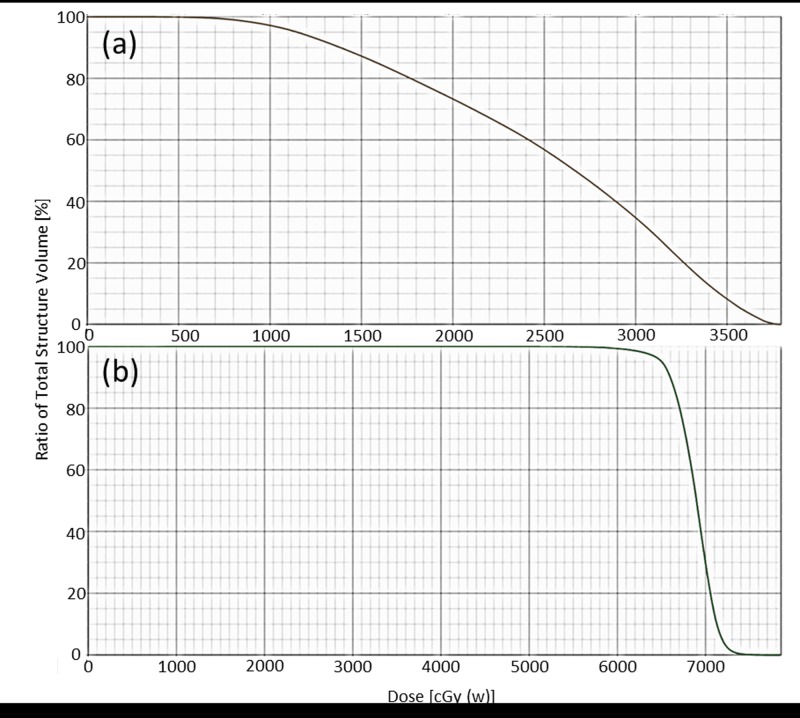
Dose volume histograms of GTV for the BNCT alone (a) and BNCT+IMRT (b) plans for a recurrent parotid gland tumor.

**Fig 4:**
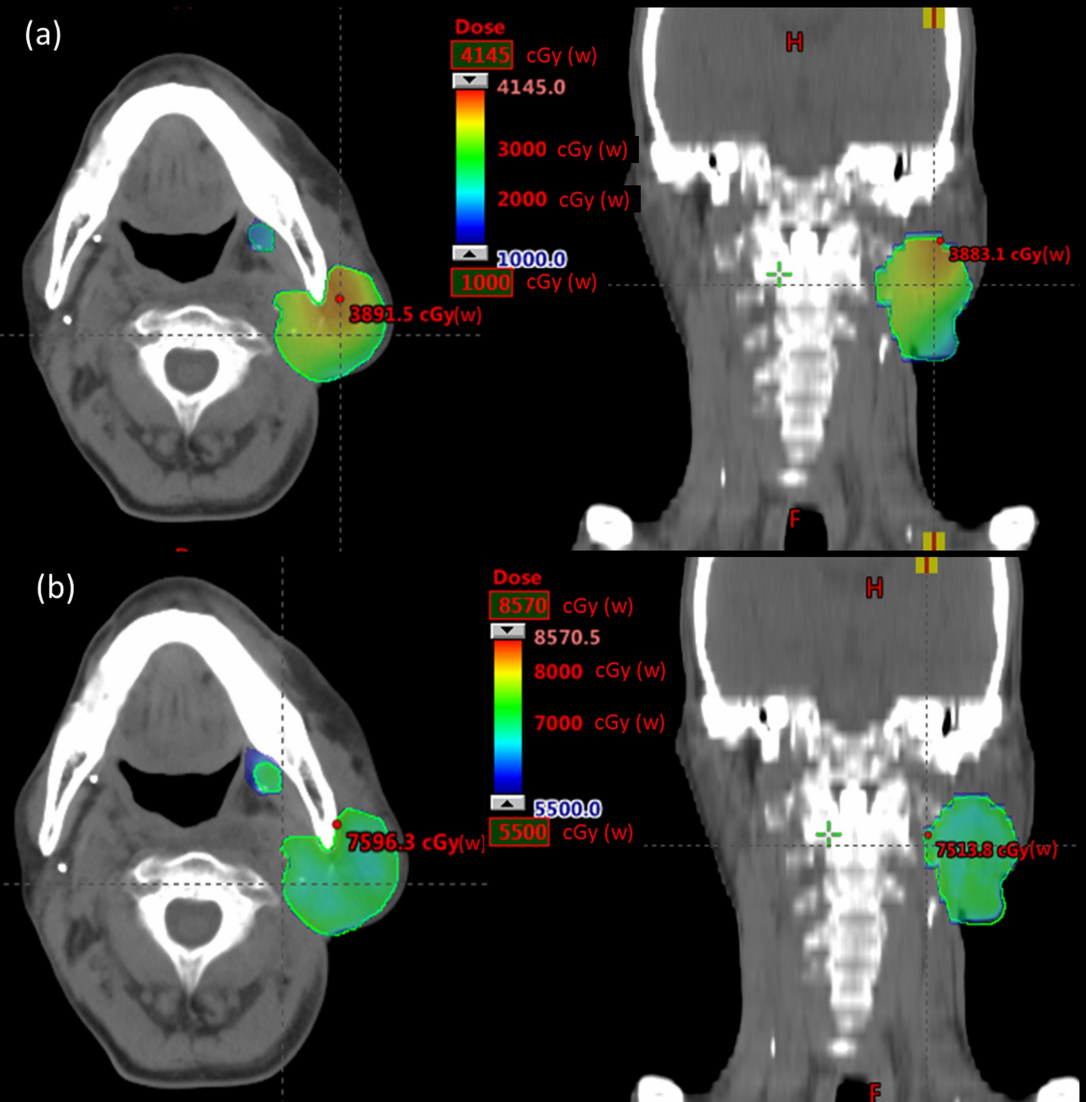
Representative isodose curves of GTV for the BNCT alone (a) in axial and coronal views and BNCT+IMRT (b) plans in axial and coronal views for a recurrent oropharyngeal cancer.

**Fig 5:**
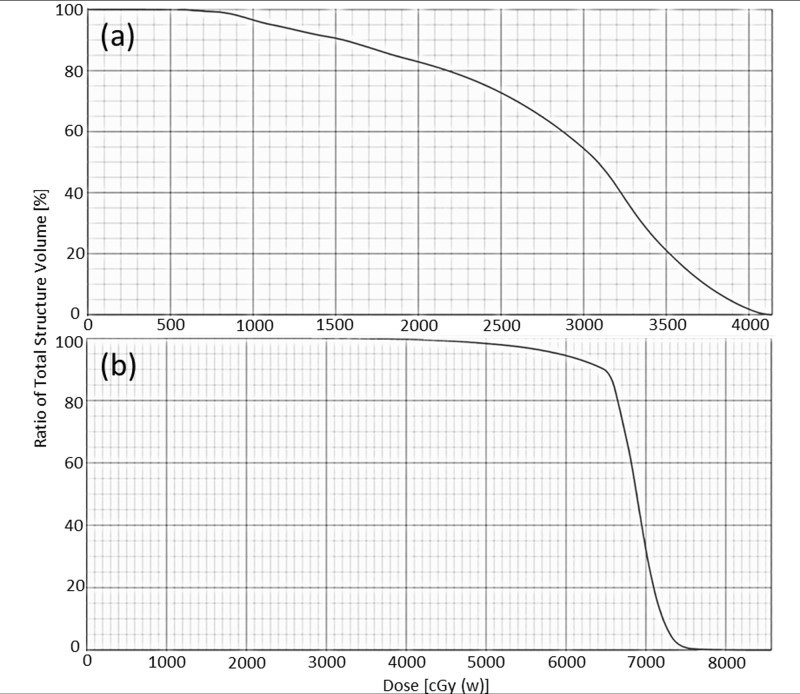
Dose volume histograms of GTV for the BNCT alone (a) and BNCT+IMRT (b) plans for a recurrent oropharyngeal cancer.

Representative dose distributions of the BNCT and BNCT+IMRT plans for two patients (case 5 & 8) with recurrent parotid gland tumor and oropharyngeal cancer are presented in Figs 2 and 3. The dose deviation for the GTV was greater in the BNCT plan than in the BNCT+IMRT plan. Dose volume histograms of the two plans for each patient are presented in Figs 4 and 5.

Mean relative volumes that represented volumes covering 110% (V_110_), 95% (V_95_), and 90% (V_90_) of the PDs for both treatment planning volumes were compared, as well as the mean dose, CI, HI, total beam-on time, and monitor units (MU), for the BNCT and BNCT+IMRT plans (Table 2). All results were shown as mean ± SD. The mean GTV was 80.7 ± 111.9 cm^3^, and the mean GTV doses in the BNCT and BNCT+IMRT plans were 130.7% and 106.2%, respectively, of the PDs. The CI value for the GTV in the BNCT+IMRT plan (0.96 ± 0.01) was significantly better than that in the BNCT plan (0.73 ± 0.13; *p* = 0.005). The BNCT+IMRT plan also provided significantly better homogeneity for the GTV than did the BNCT plan based on HI values (1.11 ± 0.05 vs. 1.72 ± 0.59; *p* = 0.005; Table 2).

**Table 2:** Parameters of the BNCT and BNCT+IMRT plans, applied to nine patients.

Treatment parameter		BNCT			BNCT+IMRT		
GTV	Mean volume (cm^3^)	81.93 ± 114.28					
	V_110_ (%)	56.94	±	24.34	11.36	±	25.59
	V_95_ (%)	75.53	±	15.80	98.38	±	0.85
	V_90_ (%)	81.37	±	12.08	99.35	±	0.76
	Mean dose (Gy (w))	23.52	±	4.66	69.03	±	1.56
	Minimum dose (Gy (w))	3.89	±	1.93	46.35	±	9.32
	CI	0.73	±	0.13	0.96	±	0.01
	HI	1.72	±	0.59	1.11	±	0.05
Total beam-on time (min)		25.97	±	4.84	27.33^a^	±	5.39
MU		-		-	818.78	±	533.19

All data are presented as the mean ± standard deviation.

^a^ Based on a summation of the beam-on times for the BNCT plan and the IMRT-alone plan (25.97 ± 4.84 and 1.37 ± 0.89 min per fraction, respectively).

BNCT, boron neutron capture therapy; IMRT, intensity-modulated radiation therapy; GTV, gross tumor volume; CI, conformity index; HI, homogeneity index; MU, monitor unit.

The GTVs in patients 2 and 5 (group 1) were >100 cm^3^, and those in the rest of the patients (group 2) were <100 cm^3^. The CI and HI values for GTV in group2 were 0.73 ± 0.14 and 1.47 ± 0.23, respectively, for the BNCT plan, and 0.96 ± 0.01 and 1.09 ± 0.02, respectively, for the BNCT+IMRT plan. In group 1, these values were 0.72 ± 0.11 and 2.61 ± 0.64, respectively, for the BNCT plan, and 0.95 ± 0.01 and 1.17 ± 0.07, respectively, for the BNCT+IMRT plan. Compared with the BNCT plan, the mean CI and HI values for group 2 were improved by 31.5% and 25.9%, respectively, in the BNCT+IMRT plan. Group 1 showed improvements of 31.9% and 57.4%, respectively. BNCT+IMRT thus improved the CI and HI to a greater degree for larger GTVs.

In the IMRT plan, the mean MU value was 818.78 ± 533.19. The daily beam-on times of the BNCT and BNCT+IMRT plans were similar because that of the IMRT-alone plan was 1.37 ± 0.89 min at a 600 MU/min dose rate, and a large proportion of the total beam-on time derived from the BNCT plan. In the IMRT plan, the total mean dose was 45.77 ± 3.66 Gy (w) and the mean dose per fraction was 2.29 ± 0.17 Gy (w). An additional consideration is that dose inhomogeneity in IMRT may impair its biological effect in each fraction. As the GTV received a mean dose > 2 Gy (w) /fraction (Table 1), our IMRT plan may be adequate for daily treatment. Our BNCT+IMRT plan provided better homogeneity than did the three-gradient XRT method described by Kawabata et al. [15], which involved the application of an additional 24 Gy (2 Gy daily × 12 fractions) XRT.

Table 3 lists the dosimetric parameters for the OAR. For the spinal cord, the maximum significant dose and physical maximum point dose were 2.33 ± 0.93 and 2.73 ± 1.07 Gy (w), respectively, in the BNCT plan, and 16.43 ± 9.85 and 21.73 ± 11.12 Gy (w), respectively, in the BNCT+IMRT plan. The mean dose, D_2/3V_, and D_1/2V_ of the parotid were 1.65 ± 1.25, 0.55 ± 0.51, and 1.77 ± 1.86 Gy (w), respectively, in the BNCT plan, and 9.54 ± 4.28, 5.94 ± 2.99, and 7.62 ± 3.99 Gy (w), respectively, in the BNCT+IMRT plan. The physical maximum point doses for the mandible were 21.81 ± 11.04 and 56.99 ± 16.57 Gy (w) in the BNCT and BNCT+IMRT plans, respectively. In order to score for PD and low dose spillage outside the PTV, the DSI_45_ for the BNCT+IMRT plan was 0.20 ± 0.19, and DSI_5_ values for the BNCT and BNCT+IMRT plans were 6.22 ± 5.81 and 54.83 ± 52.24, respectively.

**Table 3:** Metrics evaluated for the OAR in nine patients.

OAR parameter		BNCT			BNCT+IMRT				
Spinal cord	MSD (Gy (w))	2.33	±	0.93	16.43			±	9.85
	PMPD (Gy (w))	2.73	±	1.07	21.73			±	11.12
Brainstem	MSD (Gy (w))	1.76	±	0.68	11.26			±	12.19
	PMPD (Gy (w))	2.22	±	0.88	17.24			±	15.17
Parotids	Volume (cm^3^)	25.36 ± 11.19							
	Mean (Gy (w))	1.61	±	1.20	9.54			±	4.28
	D_2/3V_ (Gy (w))	0.56	±	0.51	5.94			±	2.99
	D_1/2V_ (Gy (w))	1.74	±	1.81	7.62			±	3.99
Inner Ears	Volume (cm^3^)	1.92 ± .57							
	Mean (Gy (w))	1.71	±	0.86	6.58			±	9.34
Eyes	Volume (cm^3^)	17.26 ± 1.84							
	Mean (Gy (w))	0.9	±	0.61	2.53			±	4.23
	PMPD (Gy (w))	2.31	±	1.51	5.76			±	9.18
Lens	Volume (cm^3^)	0.29 ± 0.09							
	Mean (Gy (w))	0.68	±	0.47	1.74			±	2.54
Mandible	Volume (cm^3^)	67.63 ± 23.73							
	Mean (Gy (w))	1.72	±	0.61	15.13			±	4.73
	PMPD (Gy (w))	10.51	±	2.78	56.99			±	16.57
DSI_45_		-		-	0.20			±	0.19
DSI_5_		6.22	±	5.81	54.83			±	52.24

All data are expressed as the mean ± standard deviation.OAR, organs at risk; BNCT, boron neutron capture therapy; IMRT, intensity-modulated radiation therapy; MSD, maximum significant dose; PMPD, physical maximum point dose; DSI, dose spillage index. Mean DSI of 45 Gy (w) outside of the planning target volume. Mean DSI of 5 Gy (w) outside of the gross tumor volume.

## Discussion

Re-irradiation for recurrent H & N cancer remains a therapeutic challenge due to high toxicity and low successful rate. From our previous two-fraction BNCT trial, re-recurernce within or near the GTV was common, in spite of initial good response. Combined BNCT and IMRT was tested in our 2^nd^ clinical trial to obtain better results. In this study, we searched for an “ideal” IMRT plan to well-compensate for the cold and hot spots commonly seen in BNCT alone. Theoretically, local control will be improved by this approach.

The maximum significant dose and physical maximum point dose for the spinal cord were less than our initial planning objective (< 20 Gy (w)). In addition, large proportions of the OAR doses were derived from IMRT due to the percent depth dose property of the photons and the photon beam direction. The low dose volume outside the GTV was 8.8 times larger in the BNCT+IMRT plan than in the BNCT plan due to the photon dose of the former (Table 3). The DSI_45_ value for the BNCT+IMRT plan indicates that the volume receiving 45 Gy (w) outside the PTV was small (Table 3). Based on these results, BNCT+IMRT appears to comprise a relatively good conformal plan.

Other researchers have sought to improve dose homogeneity in BNCT by combining more than one neutron field [24]. However, even with technique employing two or more fields, it remains difficult to obtain an ideal dose distribution with inhomogeneous boron drug distribution in large tumors, like those found in some of our patients. The dose distribution in the single-field BNCT plan in the present study was inhomogeneous and cold spots of dose were found in tumor, entailing the possibility of H&N cancer recurrence after BNCT alone. Because only three dose gradients were used in a Japanese combined-treatment study [15], BNCT+IMRT provided better dose distribution in the GTV; this plan may increase local tumor control further by compensating the low-dose region with daily IMRT. We thus believe our plan provide a better solution of combining BNCT and photon therapy than that offered by Kawabata’s study. However, normal tissue doses (e.g., to the mandible) may be higher in BNCT+IMRT than in BNCT alone (Table 3); low doses (5 Gy (w)) volume to the normal tissue may be much greater (Table 3). The dose inhomogeneity for the GTV in the IMRT plan may influence its biological effect. However, the daily mean dose for the GTV remained larger than 2 Gy (w)/fraction (Table 1). We thus believe that this simulated IMRT plan can be applied in clinical practice.

The limitations of current study include small case numbers and heterogeneous in stages, tumor volumes, tumor locations. More patients are needed to fairly compare BNCT and combined treatment. On the other hand, higher total dose in the combined treatment might increase toxicity. Based on our dosimetric study and limited clinical evidences, we would not recommend combined BNCT+IMRT for all recurrent H & N cancer in clinics, but this combination can be considered in selected cases with large recurrent tumors for whom higher total dose and better homogeneity is desired.

## Conclusions

In this study, we demonstrated that BNCT+IMRT may improve treatment homogeneity and conformity, as well as possible local tumor control without increasing hazardous high dose to the normal tissues.
